# Multiple long-term conditions within households and use of health and social care: a retrospective cohort study

**DOI:** 10.3399/BJGPO.2020.0134

**Published:** 2021-02-24

**Authors:** Mai Stafford, Sarah R Deeny, Kathryn Dreyer, Jenny Shand

**Affiliations:** 1 The Health Foundation, London, UK; 2 UCLPartners, London, UK; 3 Institute of Epidemiology & Health Care, University College London, London, UK; 4 Care City, Barking, UK

**Keywords:** comorbidity, multimorbidity, multiple conditions, inequalities, social context, socioeconomic factors

## Abstract

**Background:**

The daily management of long-term conditions falls primarily on individuals and informal carers, but the impact of household context on health and social care activity among people with multiple long-term conditions (MLTCs) is understudied.

**Aim:**

To test whether co-residence with a person with MLTCs (compared with a co-resident without MLTCs) is associated with utilisation and cost of primary, community, secondary health care, and formal social care.

**Design & setting:**

Linked data from health providers and local government in Barking and Dagenham for a retrospective cohort of people aged ≥50 years in two-person households in 2016–2018.

**Method:**

Two-part regression models were applied to estimate annualised use and cost of hospital, primary, community, mental health, and social care by MLTC status of individuals and co-residents, adjusted for age, sex, and deprivation. Applicability at the national level was tested using the Clinical Practice Research Datalink (CPRD).

**Results:**

Forty-eight per cent of people with MLTCs in two-person households were co-resident with another person with MLTCs. They were 1.14 (95% confidence interval [CI] = 1.00 to 1.30) times as likely to have community care activity and 1.24 (95% CI = 0.99 to 1.54) times as likely to have mental health care activity compared with those co-resident with a healthy person. They had more primary care visits (8.5 [95% CI = 8.2 to 8.8] versus 7.9 [95% CI = 7.7 to 8.2]) and higher primary care costs. Outpatient care and elective admissions did not differ. Findings in national data were similar.

**Conclusion:**

Care utilisation for people with MLTCs varies by household context. There may be potential for connecting health and community service input across household members.

## How this fits in

It is known that care utilisation and cost are higher for people with multiple long-term conditions (MLTCs) but not whether this association varies by health status of household co-residents. Greater primary care, community care, and mental health care activity were found where a person with MLTCs was co-resident with another person with MLTCs. This affirms the need to identify and support informal carers. The potential to improve how health and care services are connected across people within a household should be considered.

## 



## Introduction

Meeting the needs of people with MLTCs is a key challenge facing health and social care systems. In the UK, around 25% of people (depending on the conditions included) have ≥2 conditions and their care needs account for >50% of primary and secondary care costs, and a substantial portion of community and social care costs.^1–3^ Trials of initiatives to improve outcomes and reduce hospital or emergency care use of those with MLTCs have not shown success, at least in the short term.^4,5^ Initiatives have focused on providing patient-centred care for people with MLTCs,^6^ but if elements of care use and costs are to be reduced then more research is needed on their drivers. The potential for considering the household context in the management of ongoing conditions has so far received little attention.

The daily responsibility for managing their conditions usually falls primarily on the individuals themselves and on their informal carers. Around half of carers in England provide care for someone in the same household.^7^ However, studies of the household context and its impact on service use and cost among people with MLTCs have focused on household size ^8^ and not household members’ health status. Co-residents are at increased risk of having long-term conditions^9,10^ because of shared lifestyle,^11^ social risk factors, and the tendency to select a similar partner. A co-resident’s illness may make it more difficult for them to provide practical, financial, or emotional support.

This study examined whether co-residence with another person with MLTCs is associated with higher use and cost of primary, community, secondary health care, and formal social care. Difficulty in identifying households within electronic health records (EHRs) likely contributes to the lack of research in this area. To address this, a local sample of EHRs was used that were linked to household composition data from local authority records and the analysis was replicated in a national study where co-residence was inferred from anonymised address data.

## Method

The study included people aged ≥50 years in two-person households. The prevalence of MLTCs rises with age and the combination of conditions also varies with age, with mental and physical comorbidities being more prevalent at younger ages.^1^ The study, therefore, focuses on middle and older age, when people may be experiencing changes in health and functioning. People in households with ≥3 occupants were excluded to remove institutions or situations where there could be multiple people providing help.

### Main analytical sample: Barking and Dagenham

Linked information from local government services, health providers, and health commissioners includes sociodemographic, health, and household information alongside activity data for five care settings (primary care, hospital, community, inpatient and outpatient mental health services, and social care). Residents of Barking and Dagenham, a borough in outer London, were included from 1 April 2016 to 31 March 2018. Those who moved out of Barking and Dagenham or who died before the 1 April 2018 were excluded, given the known increase in healthcare utilisation at the end of life.^12^ Household composition was derived using the unique property reference number in local government records. The analytical sample was 9222 individuals in 4611 two-person households.

### Measuring MLTCs

Long-term conditions associated with higher treatment needs, poorer quality of life, or greater risk of premature death were included.^1,13,14^ Sixteen long-term mental and physical health conditions were identified from diagnosis codes recorded in primary care (see Supplementary Table S1). People with ≥2 conditions were classified as having 'MLTCs' and those with 0–1 condition as 'healthy'. Individuals were assigned to one of four categories: person with MLTCs co-resident with healthy person (MLTCs/healthy; the reference group); person with MLTCs co-resident with another (Both MLTCs); healthy person co-resident with healthy person (Both healthy); healthy person co-resident with person with MLTCs (healthy/MLTCs).

### Health and social care activity and cost

The following health and social care activity and cost outcomes were examined over 2 years: number and cost of primary care consultations with a GP, nurse, or other clinical staff; days in hospital and cost of hospital care (broken down into outpatient consultations, elective admissions, non-elective admissions, and emergency department [ED] attendances); cost of mental health inpatient and outpatient care; cost of community health services; and cost of local government funded social care.

Local unit level costs were used for community and mental health activity and social care services. All other costs were based on activity using national reference costs for the relevant year. Mean cost for the relevant activity was used where an activity code could not be matched to a national reference cost. Costs were indexed to 2018 prices. See Supplementary Table S2 for more detail on the costing method.

### Statistical analysis

The distributions of care costs and days in hospital show a substantial proportion of people having zero activity. Two-part mixture models were used to account for their semi-continuous distributions. The first part used a logistic model to estimate the likelihood of having any versus no care. The second part used a gamma model to estimate the cost or utilisation among the subset where this was non-zero. Sex, age group, deprivation (Index of Multiple Deprivation quintile for the patient’s residence), and MLTC status were included as covariates for both parts. Mean costs and activity across both parts of the model combined were also estimated. Individual level analysis with robust standard errors allowed for the non-independence of individuals within households.

### Replication in a national sample

Nationally representative data were obtained from the CPRD (approved protocol: ISAC17_150RMn2). CPRD comprises de-identified records of >14 million patients^15^ linked to Hospital Episode Statistics (HES) for consenting practices in England. Individuals were included who were registered in up-to-standard practices (derived by CPRD using information on continuity of recording and number of recorded deaths) from 1 April 2014 to 31 March 2016.

The CPRD pseudonymised family number (based on the first line of the patient’s address) was used to select a sample where exactly two patients shared a family identifier and were registered within 1 year of each other. This excludes households where members are not registered at the same GP practice. From an initial random sample of 300 000 children and adults with linked HES data, 10 528 met inclusion criteria and formed the analytical sample for this study.

The presence of 36 mental and physical health conditions recorded in primary care^16^ was determined on 1 April 2014 based on diagnosis (using Read codes) and prescribing data (see Supplementary Table S3). There is considerable overlap with the 16 broader groups of long-term conditions included in the main analysis. GP and hospital care activity and costs were calculated using the same approach but mental health care, community health care, and social care data were not available.

## Results

### Sample description: main sample

The prevalence of MLTCs was 43% for those aged ≥50 years living in two-person households in Barking and Dagenham. Forty-eight per cent of people with MLTCs lived with another person with MLTCs (Table 1). Older people and those in deprived areas were over-represented in households with two residents with MLTCs.

**Table 1.** Barking and Dagenham and Clinical Practice Research Datalink sample characteristics

	MLTCs/healthy co-resident	Both MLTCs	Healthy/MLTCs co-resident	Both healthy	All participants
	***n***	**row %**	***n***	**row %**	***n***	**row %**	***n***	**row %**	***n***	**col %**
**B & D sample**	2049	22.2	1884	20.4	3240	35.1	2049	22.2	9222	100.0
**Age** **, years**										
50–59	337	14.4	205	8.8	1240	53.1	554	23.7	2336	25.3
60–69	620	20.9	566	19.1	1124	37.9	655	22.1	2965	32.2
70–79	644	26.0	663	26.8	595	24.0	575	23.2	2477	26.9
≥80	448	31.0	450	31.2	281	19.5	265	18.4	1444	16.7
**Sex**										
Male	1044	22.7	928	20.1	1640	35.6	997	21.6	4609	50.0
Female	1005	21.8	956	20.7	1600	34.7	1052	22.8	4613	50.0
**IMD quintile**										
1 (least deprived)	0	0	0	0	0	0	0	0	0	0
2	0	0	0	0	0	0	0	0	0	0
3	192	21.7	163	18.4	339	38.3	192	21.7	886	9.6
4	768	22.5	617	18.1	1263	37.0	770	22.5	3418	37.1
5 (most deprived)	1089	22.1	1104	22.4	1638	33.3	1087	22.1	4918	53.3
**Carer code**										
Present	134	31.2	158	36.8	81	18.9	56	13.1	429	4.7
Not present	1915	21.8	1726	19.6	3159	35.9	1993	22.7	8793	95.3
**CPRD sample**	2107	20.0	2940	27.9	3374	32.0	2107	20.0	10 528	100.0
**Age** **, years**										
50–59	530	15.8	372	11.1	1770	52.7	687	20.5	3359	31.9
60–69	791	21.4	915	24.7	1179	31.8	817	22.1	3702	35.2
70–79	568	22.7	1081	43.3	377	15.1	471	18.9	2497	23.7
≥80	218	22.5	572	59.0	48	4.9	132	13.6	970	9.2
**Sex**										
Male	1171	22.3	1464	27.8	1697	32.3	928	17.6	5260	50.0
Female	936	17.8	1476	28.0	1677	31.8	1179	22.4	5268	50.0
**IMD quintile**										
1 (least deprived)	728	20.0	871	24.0	1306	35.9	729	20.1	3634	34.5
2	491	19.8	674	27.1	825	33.2	496	20.0	2586	24.6
3	426	20.2	598	28.3	662	31.4	425	20.1	2111	20.1
4	302	19.9	510	33.6	410	27.0	296	19.5	1518	14.4
5 (most deprived)	160	20.5	287	36.8	171	22.0	161	20.7	779	7.4
**Carer code**										
Present	48	12.4	197	52.7	48	12.4	81	21.7	374	3.6
Not present	2059	20.3	2743	27.0	3326	32.8	2026	20.0	10154	96.4

B & D = Barking and Dagenham. CPRD = Clinical Practice Research Datalink. IMD = Index of Multiple Deprivation. MLTCs = multiple long-term conditions.

Over 97% of people had at least one primary care consultation, but over 30% had no outpatient attendance, 80% had no emergency department attendance, and over 80% had no inpatient admission (see Supplementary Table S4).

### Model results: main sample

The association between each person’s household MLTC status and the likelihood of having any care activity is shown in Table 2 (see column labelled 'OR for any activity'). The focus is on people with MLTCs co-resident with a person with or without MLTCs. Controlling for sex, age, and socioeconomic deprivation, the Both MLTCs group was 1.14 (95% CI = 1.00 to 1.30) times as likely to have any community care activity. In addition, the Both MLTCs group was 1.24 (95% CI = 0.99 to 1.54) times as likely to have any mental health care activity, and 1.24 (95% CI = 0.96 to 1.59) times as likely to have any social care activity as the MLTCs/healthy reference group.

**Table 2.** Two-part models for health and social care activity: Barking and Dagenham sample of *n* = 9222 people aged ≥50 years in two-person households

	OR for any activity	95% LL	95%UL	Exp gamma coefficient (in the subset with any activity)	95% LL	95% UL	Adjusted means across both parts	95% LL	95% UL
**Number of primary care visits**									
MLTCs/healthy	Reference			Reference			7.9	7.7	8.2
Both MLTCs	0.44	0.17	1.11	1.08	1.03	1.12	8.5	8.2	8.8
Both healthy	0.12	0.06	0.27	0.62	0.60	0.65	4.8	4.7	4.9
Healthy/MLTCs	0.13	0.06	0.30	0.66	0.64	0.69	5.1	5.0	5.3
**Primary care costs**									
MLTCs/healthy	Reference			Reference			£198	£192	£205
Both MLTCs	0.44	0.17	1.11	1.08	1.03	1.13	£213	£205	£220
Both healthy	0.12	0.06	0.27	0.64	0.62	0.67	£124	£121	£128
Healthy/MLTCs	0.13	0.06	0.30	0.69	0.66	0.72	£134	£130	£138
**Outpatient costs**									
MLTCs/healthy	Reference			Reference			£312	£294	£330
Both MLTCs	1.03	0.89	1.19	0.93	0.86	1.00	£293	£275	£311
Both healthy	0.50	0.44	0.56	0.71	0.66	0.76	£172	£162	£182
Healthy/MLTCs	0.51	0.45	0.58	0.73	0.68	0.79	£181	£168	£193
**Cost of non-elective admissions**									
MLTCs/healthy	Reference			Reference			£609	£532	£686
Both MLTCs	1.01	0.87	1.17	0.99	0.85	1.14	£606	£527	£684
Both healthy	0.45	0.39	0.53	0.76	0.64	0.90	£247	£205	£288
Healthy/MLTCs	0.42	0.35	0.50	0.63	0.53	0.76	£192	£154	£230
**Cost of elective admissions**									
MLTCs/healthy	Reference			Reference			£443	£396	£491
Both MLTCs	1.05	0.92	1.20	1.00	0.88	1.14	£459	£409	£509
Both healthy	0.57	0.51	0.65	0.91	0.80	1.03	£273	£244	£303
Healthy/MLTCs	0.64	0.56	0.73	0.86	0.75	0.98	£281	£245	£316
**ED costs**									
MLTCs/healthy	Reference			Reference			£84	£76	£92
Both MLTCs	1.08	0.95	1.23	1.04	0.93	1.16	£91	£82	£99
Both healthy	0.53	0.47	0.60	0.76	0.67	0.85	£43	£38	£47
Healthy/MLTCs	0.52	0.45	0.59	0.78	0.68	0.88	£43	£38	£48
**Total hospital costs (outpatient** **+** **admissions** **+** **ED** **)**									
MLTCs/healthy	Reference			Reference			£1454	£1338	£1570
Both MLTCs	1.06	0.91	1.24	0.99	0.89	1.11	£1458	£1338	£1578
Both healthy	0.49	0.43	0.56	0.62	0.56	0.69	£743	£685	£800
Healthy/MLTCs	0.47	0.41	0.54	0.59	0.53	0.67	£701	£636	£766
**Number of days in hospital**									
MLTCs/healthy	Reference			Reference			1.58	1.33	1.83
Both MLTCs	0.97	0.84	1.12	0.98	0.80	1.20	1.53	1.28	1.77
Both healthy	0.43	0.37	0.50	0.80	0.63	1.00	0.65	0.51	0.78
Healthy/MLTCs	0.42	0.35	0.50	0.67	0.52	0.87	0.54	0.41	0.67
**Community care costs**									
MLTCs/healthy	Reference			Reference			£428	£358	£498
Both MLTCs	1.14	1.00	1.30	1.20	0.97	1.49	£559	£468	£650
Both healthy	0.37	0.32	0.43	0.69	0.54	0.89	£141	£111	£171
Healthy/MLTCs	0.36	0.31	0.42	0.51	0.39	0.68	£103	£77	£128
**Mental health costs**									
MLTCs/healthy	Reference			Reference			£318	£155	£482
Both MLTCs	1.24	0.99	1.54	1.41	0.73	2.71	£543	£281	£795
Both healthy	0.37	0.28	0.50	1.01	0.43	2.40	£127	£31	£224
Healthy/MLTCs	0.40	0.30	0.54	0.76	0.30	1.92	£103	£19	£186
**Social care costs**									
MLTCs/healthy	Reference			Reference			£439	£320	£559
Both MLTCs	1.24	0.96	1.59	0.86	0.66	1.13	£461	£347	£575
Both healthy	0.29	0.20	0.43	0.58	0.38	0.88	£80	£42	£118
Healthy/MLTCs	0.27	0.18	0.41	0.51	0.32	0.81	£64	£28	£99

ED = emergency department. Exp = exponentiated. LL= lower limit. MLTCs = multiple long-term conditions. OR = odds ratio. UL = upper limit.

**Table 3.** Two-part models for healthcare activity: Clinical Practice Research Datalink sample of *n* = 10 528 people aged ≥50 years in two-person households

	OR for any activity	95 %LL	95 %UL	Exp gamma coefficient (in the subset with any activity)	95 %LL	95 %UL	Adjusted means across both parts	95 %LL	95 %UL
**Number of primary care visits**										
MLTCs/healthy	Reference			Reference			9.6	9.2	9.9	
Both MLTCs	1.31	0.67	2.56	1.09	1.03	1.15	10.5	10.1	10.8	
Both healthy	0.09	0.06	0.14	0.49	0.47	0.52	4.3	4.2	4.5	
Healthy/MLTCs	0.12	0.08	0.19	0.56	0.53	0.59	5.0	4.8	5.2	
**Primary care costs**										
MLTCs/healthy	Reference			Reference			£247	£237	£258	
Both MLTCs	1.31	0.67	2.56	1.13	1.07	1.19	£280	£269	£290	
Both healthy	0.09	0.06	0.14	0.52	0.49	0.55	£117	£112	£123	
Healthy/MLTCs	0.12	0.08	0.19	0.55	0.52	0.59	£128	£122	£134	
**Outpatient costs**										
MLTCs/healthy	Reference			Reference			£483	£443	£523	
Both MLTCs	1.09	0.95	1.25	1.01	0.92	1.11	£498	£468	£528	
Both healthy	0.35	0.31	0.39	0.69	0.60	0.79	£222	£194	£250	
Healthy/MLTCs	0.41	0.37	0.47	0.68	0.58	0.79	£239	£207	£271	
**Cost of non-elective admissions**										
MLTCs/healthy	Reference			Reference			£294	£244	£345	
Both MLTCs	1.19	1.02	1.39	1.04	0.87	1.24	£351	£302	£401	
Both healthy	0.40	0.33	0.49	0.84	0.66	1.08	£111	£84	£139	
Healthy/MLTCs	0.47	0.38	0.57	0.85	0.68	1.05	£128	£99	£157	
**Cost of elective admissions**										
MLTCs/healthy	Reference			Reference			£599	£525	£673	
Both MLTCs	1.16	1.03	1.31	0.89	0.79	0.99	£587	£528	£645	
Both healthy	0.50	0.43	0.57	0.72	0.63	0.82	£255	£218	£292	
Healthy/MLTCs	0.54	0.47	0.62	0.70	0.62	0.79	£266	£224	£307	
**ED costs**										
MLTCs/healthy	Reference			Reference			£69	£62	£76	
Both MLTCs	1.11	0.98	1.26	1.11	0.99	1.25	£82	£74	£89	
Both healthy	0.53	0.47	0.61	0.76	0.67	0.87	£33	£29	£37	
Healthy/MLTCs	0.56	0.49	0.64	0.73	0.65	0.81	£33	£29	£36	
**Total hospital costs (outpatient** **+** **admissions** **+** **ED** **)**										
MLTCs/healthy	Reference			Reference			£1445	£1314	£1576	
Both MLTCs	1.10	0.94	1.29	1.03	0.93	1.15	£1525	£1418	£1630	
Both healthy	0.33	0.29	0.38	0.62	0.54	0.71	£631	£558	£703	
Healthy/MLTCs	0.38	0.34	0.44	0.45	0.39	0.52	£664	£589	£739	
**Number of days in hospital**										
MLTCs/healthy	Reference			Reference			0.91	0.72	1.11	
Both MLTCs	1.08	0.94	1.24	0.89	0.70	1.12	0.86	0.73	0.99	
Both healthy	0.36	0.30	0.43	0.83	0.61	1.12	0.31	0.23	0.40	
Healthy/MLTCs	0.42	0.35	0.51	0.66	0.50	0.86	0.29	0.22	0.36	

ED = emergency department. Exp = exponentiated. LL = lower limit. MLTC = multiple long-term conditions. OR = odds ratio. UL = upper limit.

For those with any activity, exponentiated gamma coefficients show the association between household MLTC status and level of care activity or cost. The coefficient of 1.08 (95% CI = 1.03 to 1.12) for primary care visits shows the number of visits for the Both MLTCs group was 8% (95% CI = 3% to 12%) higher than the reference group. A similar difference in cost of primary care visits was seen. There was no evidence of difference in costs between these groups for other outcomes.

**Figure 1.**
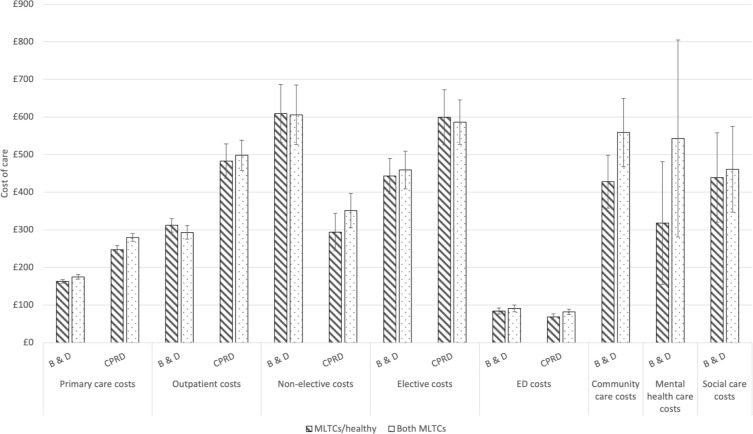
Cost of care by household multimorbidity status. B & D = Barking and Dagenham. CPRD = Clinical Practice Research Datalink. ED = emergency department. MLTCs = multiple long-term conditions.

The adjusted means combine estimates from both parts of the model, including those with and without the relevant activity. Adjusted mean annual primary care costs and number of visits were higher for the Both MLTCs group than the reference. Community care costs for the Both MLTCs group were also higher (£559 versus £428), as were mental health care costs (£543 versus £318), although CIs overlapped (Figure 1).

Although not the focus of this study, those without MLTCs (in the Both healthy or the Healthy/MLTCs group) were less likely to have any activity than the reference group and had lower adjusted mean costs for all types of care.

### Replication in the national sample

The national sample was younger than the main sample (32% versus 25% aged 50–59 years) and less socioeconomically deprived (Table 1). The prevalence of MLTCs was 48% in the national sample and 58% of people with MLTCs were co-resident with another person with MLTCs. The Both MLTCs group was more likely to have a non-elective hospital admission (odds ratio [OR] = 1.19 [95% CI = 1.02 to 1.39]) or elective admission and somewhat more likely to have any ED visit (OR = 1.11 [95% CI = 0.98 to 1.26]) compared with the reference group (Table 3).

In the subset with non-zero cost or activity, primary care costs were 13% (95% CI = 7% to 19%) higher in the Both MLTCs group compared with the reference, and the Both MLTCs group also had more primary care visits. There was a suggestion of higher ED costs (exponentiated gamma coefficient 1.11 [95% CI = 0.99 to 1.25, *P* = 0·06]) with no evidence of difference in costs between these groups for other outcomes.

As in the local sample, combined estimates across both parts of the model showed significantly higher adjusted primary care costs and number of visits for the Both MLTCs group (10.5 visits) than the reference (9.6 visits).

## Discussion

### Summary

This study shows that the household context matters for the care use of people with MLTCs, independently of age, sex, and area deprivation. Evidence from one local and one national sample showed higher primary care costs and visits where two people with MLTCs were co-resident. Where data were available (in the local sample only), these showed a greater likelihood of using community care activity and a trend towards greater likelihood of using mental health care where two people with MLTCs were co-resident. Differences in secondary care according to the co-resident’s MLTC status were inconsistent in direction in the local and national datasets, although both showed a trend to slightly higher ED costs where two people with MLTCs were co-resident.

### Strengths and limitations

Two-part models were used to model cost and utilisation outcomes. Outcomes were based on EHRs and not subject to recall or reporting bias. Two large samples were used to test the hypothesis, with similar prevalence of own and co-resident MLTCs. The findings were able to be replicated in a national sample for primary care. This provides reassurance that the pseudonymised variable based on the first line of the patient’s last known address in combination with current registration date is a reasonable approach to identifying two-person households in primary care records.

While novel data linkage allowed the examination of mental health, community, and social care in Barking and Dagenham, these data are not available nationally. Replication in other local sites is needed to assess whether higher levels of community and mental health care use where both people in a two-person household have MLTCs are seen in other contexts.

Partners could not be distinguished from other types of co-resident as partnership status is infrequently recorded in EHRs.^17^ The influence of a partner may be different from that of a non-intimate co-resident, although short-term co-residents are probably rare among those aged ≥50 years. Neither could the authors identify informal caregivers because this also is not well recorded in administrative records. In this study, <5% had a Read code relating to caring whereas the 2011 census found over 17% of people aged ≥50 years in England were providing informal care for someone with a long-term physical or mental health condition or disability.^18^

The number or severity of conditions or acquisition of new conditions through follow-up were not considered, although the adjustment for age and socioeconomic deprivation may partly capture this. Different lists of conditions were used in the two datasets, but there was considerable overlap and the main comparison of interest for this study was within-dataset differences between the Both MLTCs and reference groups. These findings should not be extrapolated to younger households, although household health context has previously been associated with children’s healthcare use.^19^

### Comparison with existing literature

Although unobserved confounding cannot be excluded, there are three substantive mechanisms that could underlie observed associations. These are tentative and were not directly tested in this study. First, a co-resident with MLTCs may have less capacity to provide informal support than a healthy co-resident. Lack of informal support has sometimes been associated with greater healthcare utilisation.^20^ The substitution of informal and formal care may depend on the specific type of healthcare utilisation. A study based in the US found evidence of substitution for home health, nursing home care, and physician visits but not for secondary care.^21^ Community care investigated in the present study could be considered broadly equivalent to home health care in the US, in which case the finding that community care and primary care costs varied according to the co-resident’s health status, but hospital costs did not, shows alignment with the US study if co-residents' health status is an indicator of informal support provision. Researchers have proposed that informal care and hospital care are different products so substitution of one for the other would not be expected.^21^

A second plausible explanation is that a co-resident with MLTCs has more information about available health services based on the management of their own long-term conditions than one who does not have MLTCs. There is evidence that caregivers can act as advocates in ways that increase some types of care use, by helping a patient overcome denial about their need for care or being more proactive in seeking help,^21^ although it is not known whether this advocacy role depends on the caregiver’s health status.

A third possible explanation is that the person with MLTCs may be providing care for their co-resident with MLTCs. Caregiving can be a stressful experience with negative health consequences, especially in the context of low levels of formal care,^22^ and this could contribute to increased use of health care, although carers have reported greater difficulty accessing primary care compared with non-carers,^23^ and may avoid treatment because of their caring responsibilities.^24^ This third explanation is supported by the data in the present study showing that a healthy person co-resident with a person with MLTCs also had higher primary care costs and more primary care visits than their counterparts co-resident with a healthy person.

### Implications for research and practice

The largest cost differences between a person with MLTCs co-resident with a healthy person and one co-resident with another person with MLTCs were for community care and mental health care. However, most people did not have any activity for these services and consequently, confidence intervals were wide. It was not possible to replicate this in CPRD and this highlights the need for programmes to facilitate national linkage of primary, secondary, community, and social care.^25^

The findings raise questions about how to deliver health and social care that acknowledges the household context for people with MLTCs. This could include more systematic use of information about the health status of patients and their co-residents for efficient scheduling of community care for people with MLTCs, potentially reducing the total number of visits to a household that would be needed and enabling community care practitioners to increase the amount of time spent with patients in their home. It could also include developing healthcare initiatives to households based on the principles of the group care approach.^26^ In group consultations, multiple patients see ≥1 clinicians together. This combines features of the clinician–patient consultation with features of patient support groups,^27^ and has the potential to increase available consultations and peer support. If this is to be achieved, then household context data will need to be made available to service providers within integrated care systems. This will require information governance standards to be upheld while at the same time ensuring household data can be shared for patient and public benefit.

The findings reaffirm the importance of identifying and supporting those who receive and/or provide informal care. Although carer status was not able to be examined, the high level of MLTCs within households (around 50% of patients with MLTCs in these samples are living with another person with MLTCs) suggests that some carers may be managing complex care for themselves and their care recipients. The NHS Long Term Plan^28^ and other government initiatives^29^ commit to better identifying and supporting carers, and the present study suggests this has the potential to benefit people living with MLTCs as both givers and recipients of informal care.

The number of people with MLTCs is rising and the study suggests that MLTCs may cluster in households. These trends potentially impact on care systems, notably primary care and community care, and treatment burden for patients. In addition to preventive measures to modify risk factors that are common within households,^30^ research is needed to test whether connecting service input across household members could lead to efficiency savings for health and care service providers, or reduce treatment burden for those living with MLTCs.

## References

[1] Cassell A, Edwards D, Harshfield A (2018). The epidemiology of multimorbidity in primary care: a retrospective cohort study. Br J Gen Pract.

[2] Stafford M, Steventon A, Thorlby R (2018). Understanding the health care needs of people with multiple health conditions. https://www.health.org.uk/publications/understanding-the-health-care-needs-of-people-with-multiple-health-conditions.

[3] Kasteridis P, Street A, Dolman M (2014). The importance of multimorbidity in explaining utilisation and costs across health and social care settings: evidence from South Somerset's Symphony Project. http://eprints.whiterose.ac.uk/136185/1/CHERP96_multimorbidity_utilisation_costs_health_social_care.pdf.

[4] Lloyd T, Brine R, Pearson R (2018). The impact of integrated care teams on hospital use in North East Hampshire and Farnham. Consideration of findings from the Improvement Analytics Unit. https://www.health.org.uk/publications/impact-integrated-care-teams-hospital-use-north-east-hampshire-and-farnham.

[5] Sherlaw-Johnson C, Crump H, Arora S (2018). Patient-centred care for older people with complex needs: evaluation of a new care model in outer east London. https://www.nuffieldtrust.org.uk/research/patient-centred-care-for-older-people-with-complex-needs.

[6] Salisbury C, Man M-S, Bower P (2018). Management of multimorbidity using a patient-centred care model: a pragmatic cluster-randomised trial of the 3D approach. Lancet.

[7] Health and Social Care Information Centre (2010). Survey of carers in households 2009/10. https://files.digital.nhs.uk/publicationimport/pub02xxx/pub02200/surv-care-hous-eng-2009-2010-rep1.pdf.

[8] Hopman P, Heins MJ, Korevaar JC (2016). Health care utilization of patients with multiple chronic diseases in the Netherlands: differences and underlying factors. Eur J Intern Med.

[9] Meyler D, Stimpson JP, Peek MK (2007). Health concordance within couples: a systematic review. Soc Sci Med.

[10] Patel SA, Dhillon PK, Kondal D (2017). Chronic disease concordance within Indian households: a cross-sectional study. PLoS Med.

[11] Campbell P, Shraim M, Jordan KP, Dunn KM (2016). In sickness and in health: a cross-sectional analysis of concordance for musculoskeletal pain in 13,507 couples. Eur J Pain.

[12] Payne G, Laporte A, Deber R, Coyte PC (2007). Counting backward to health care's future: using time-to-death modeling to identify changes in end-of-life morbidity and the impact of aging on health care expenditures. Milbank Q.

[13] Barnett K, Mercer SW, Norbury M (2012). Epidemiology of multimorbidity and implications for health care, research, and medical education: a cross-sectional study. Lancet.

[14] Payne RA, Mendonca SC, Elliott MN (2020). Development and validation of the Cambridge multimorbidity score. CMAJ.

[15] Herrett E, Gallagher AM, Bhaskaran K (2015). Data resource profile: Clinical Practice Research Datalink (CPRD). Int J Epidemiol.

[16] University of Cambridge, Department of Public Health and Primary Care CPRD at Cambridge — code lists. http://www.phpc.cam.ac.uk/pcu/cprd_cam/codelists.

[17] Jain A, van Hoek AJ, Walker JL (2017). Identifying social factors amongst older individuals in linked electronic health records: an assessment in a population based study. PLoS One.

[18] nomis LC3301EW Provision of unpaid care by general health by sex by age. https://www.nomisweb.co.uk/census/2011/lc3301ew.

[19] Dreyer K, Williamson RAP, Hargreaves DS (2018). Associations between parental mental health and other family factors and healthcare utilisation among children and young people: a retrospective, cross-sectional study of linked healthcare data. BMJ Paediatr Open.

[20] Babitsch B, Gohl D, von Lengerke T (2012). Re-revisiting Andersen's behavioral model of health services use: a systematic review of studies from 1998–2011. Psychosoc Med.

[21] Van Houtven CH, Norton EC (2004). Informal care and health care use of older adults. J Health Econ.

[22] Wagner M, Brandt M (2018). Long-Term care provision and the well-being of spousal caregivers: an analysis of 138 European regions. J Gerontol B Psychol Sci Soc Sci.

[23] Thomas GPA, Saunders CL, Roland MO, Paddison CAM (2015). Informal carers' health-related quality of life and patient experience in primary care: evidence from 195,364 carers in England responding to a national survey. BMC Fam Pract.

[24] Tommis Y, Robinson CA, Seddon D (2009). Carers with chronic conditions: changes over time in their physical health. Chronic Illn.

[25] NHS England, Local Government Association Local health and care record exemplars: a summary. https://www.england.nhs.uk/wp-content/uploads/2018/05/local-health-and-care-record-exemplars-summary.pdf.

[26] Edelman D, McDuffie JR, Oddone E (2012). Shared medical appointments for chronic medical conditions: a systematic review. https://www.hsrd.research.va.gov/publications/esp/shared-med-appt.pdf.

[27] Jones T, Darzi A, Egger G (2019). Process and systems: a systems approach to embedding group consultations in the NHS. Future Healthc J.

[28] NHS England (2019). The NHS Long Term Plan. www.longtermplan.nhs.uk.

[29] Department of Health and Social Care (2018). Carers action plan 2018 to 2020: supporting carers today. https://www.gov.uk/government/publications/carers-action-plan-2018-to-2020.

[30] The Academy of Medical Sciences (2015). Multiple morbidities as a global health challenge. https://acmedsci.ac.uk/file-download/38330-567965102e84a.pdf.

